# Tyrosine kinase inhibitors reprogramming immunity in renal cell carcinoma: rethinking cancer immunotherapy

**DOI:** 10.1007/s12094-017-1657-7

**Published:** 2017-04-13

**Authors:** L. M. A. Aparicio, I. P. Fernandez, J. Cassinello

**Affiliations:** 1 Medical Oncology Department, Hospital Universitario A Coruña, Xubias s/n, 15615 La Coruña, Spain; 2 Medical Oncology Department, Hospital Universitario de Cabueñes, Gijón, Spain; 3 Medical Oncology Department, Hospital Universitario de Guadalajara, Guadalajara, Spain

**Keywords:** Tyrosine kinase inhibitors, Immunomodulation, Angiogenesis, Renal cell carcinoma, Immunotherapy

## Abstract

The immune system regulates angiogenesis in cancer by way of both pro- and antiangiogenic activities. A bidirectional link between angiogenesis and the immune system has been clearly demonstrated. Most antiangiogenic molecules do not inhibit only VEGF signaling pathways but also other pathways which may affect immune system. Understanding of the role of these pathways in the regulation of immunosuppressive mechanisms by way of specific inhibitors is growing. Renal cell carcinoma (RCC) is an immunogenic tumor in which angiogenesis and immunosuppression work hand in hand, and its growth is associated with impaired antitumor immunity. Given the antitumor activity of selected TKIs in metastatic RCC (mRCC), it seems relevant to assess their effect on the immune system. The confirmation that TKIs improve cell cytokine response in mRCC provides a basis for the rational combination and sequential treatment of TKIs and immunotherapy.

## Introduction

Angiogenesis, which is regulated by a fine balance between pro- and antiangiogenic signals, represents a key event in the development of tumors. In the absence of oxygen in the tumor nucleus, expression of some transcriptional factors, such as hypoxia inducible factor (HIF), is induced. HIF enhances the expression of pro-angiogenic factors such as vascular endothelial growth factor (VEGF) and platelet-derived growth factor (PDGF). VEGF is an important inducer of angiogenesis, the expression of which is also controlled by different oncoproteins such as epidermal growth factor (EGF), K-ras, and PDGF, among others [1, 2].

Immune dysfunction has been well documented in cancer patients, including those affected by renal cell carcinoma (RCC) [3–5]. RCC patients present a shift from a type-1-mediated CD4^+^ T cell response producing interferon gamma (IFNγ) to a type-2 cytokine response, involving interleukins (IL) 4, 5, and 10. Type-1-mediated CD4^+^ T cell response is critical for the development of effective antitumor immunity, while type-2 cytokine response typically mediates humoral immunity. More specifically, tumor-specific T cell response to tumor-associated antigens MAGE-6 and EphA2 is characterized by a predominance of T cells synthesizing IL5 and IL4, together with reduced levels or a complete absence of T lymphocytes expressing IFNγ [6, 7]. However, the diminished type-1 response in RCC patients is not limited to MAGE-6 and EphA2-specific CD4^+^ T cells. It has also been reported that after undergoing primary tumor excision and/or immunotherapy and presenting a disease-free period, IFNγ-producing type-1 CD4^+^ T cells prevail in RCC patients, suggesting that tumor environment may promote a type-2 response [6]. In advanced stages of RCC, the peripheral blood lymphocyte response also changes from predominantly type 1 to type 2 after polyclonal activation [8].

Antiangiogenic molecules can inhibit many immunosuppressive mechanisms, such as regulatory T (Treg) cells, myeloid-derived suppressor cells (MDSCs), immunosuppressive cytokines, and others. Besides, they play a crucial role in inducing an efficient immunostimulatory antitumor response. In this respect, emerging evidence indicates that tyrosine kinase inhibitors (TKIs) modulate hematopoiesis and immune functions [9], and their effect on myelopoiesis depends on their different selectivity for c-kit and FLT3 receptors, expressed on hematopoietic stem cells and precursor cells [9, 10].

## Proangiogenic factors

When proangiogenic factors are induced by hypoxia or oncoproteins, the balance between pro- and antiangiogenic factors is deregulated, resulting in proliferation and migration of vascular cells and the formation of new blood vessels. The structure of new blood vessels is altered, resulting in distorted and enlarged vessels, increased permeability, irregular blood flow, and microhemorrhages in the tumor. This deregulation also leads to reduced lymphocyte infiltration in the tumor [11]. Some of these proangiogenic molecules, such as VEGF, placental growth factor (PlGF), and hepatocyte growth factor (HGF) are able to modulate immunity [12, 13].

VEGF-A is also involved in the induction of tumor immunosuppression at different levels. First, tumor-derived VEGF-A can inhibit transcription nuclear factor-κB (NF-κB) via VEGFR-1 signaling and thereby prevent dendritic cell (DC) maturation [13, 14]. In cancer patients, increases in VEGF plasma levels are also correlated with the presence of immature DCs and immature myeloid cells in peripheral blood [15]. In tumor-bearing mice and metastatic colorectal cancer patients, VEGF-A can directly activate Treg cell proliferation in a VEGFR-2-dependent manner [16], as well as contributing to tumor-associated macrophage (TAM) development, by inducing the recruitment of monocytes/macrophages to the tumor. It has also been reported that VEGF-A administration decreases the proportion and number of splenic T cells and suppresses their function [17].

PlGF prevents DC differentiation [13] and inhibits the capacity of human myeloid-derived DCs to stimulate a Th1 response, as demonstrated in some in vitro experiments [18]. HGF, produced by a large number of tumors, such as carcinomas, soft tissue sarcoma, and hematopoietic malignancies, is implicated in tumor angiogenesis [19], while its receptor c-met is not only expressed by diverse tumor cells, but also present on the surface of immune cells such as DCs [20].

## Immunosuppressive cells

### Myeloid-derived suppressor cells

MDSCs are immature myeloid cells that, in chronic inflammatory conditions, fail to eventually differentiate into granulocytes, macrophages or DCs [21, 22]. MDSCs comprise a very heterogeneous population that can present widely distinct phenotypical characteristics [23–25], although they always exhibit remarkable immunosuppressive and tumorigenic activities [23–26].

Two subsets of human MDSCs can be distinguished: granulocytic MDSCs (Lin-HLA-DR-CD33^+^ or CD11b^+^ CD14-CD15^+^) and monocytic MDSCs (14^+^HLA-DR^neg/lo^ or CD11b^+^ CD14^+^HLD-DR^neg/lo^) [24–27].

MDSC tumorigenic activity includes the secretion of angiogenic factors promoting neoangiogenesis [28], the production of growth factors, matrix metalloproteinases and cytokines that activate Th2 type and Treg cells [29–31] and the deprivation of arginine and cysteine necessary for T cell functions [25, 32]. MDSCs also enhance the production of nitric oxide and reactive oxygen species, causing T cell receptor (TCR) nitration or T cell apoptosis [25], the expression of membrane-bound transforming growth factor beta 1 (TGF-β1), inducing anergy of immune effector cells [25, 33] and a down-regulation of the TCR-chain expression, disabling the capacity of T cells to transmit activation signals [25].

In summary, MDSCs create favorable conditions for tumorigenesis, tumor growth, metastasis, and neoangiogenesis, and confer tumor resistance to antiangiogenic drugs [34]. These processes are tightly interrelated and are governed by MDSC-derived mediators, such as apoptotic factors (TNF-α), interleukins (IL-1, IL-6), growth factors [TGF-β, VEGF, basic fibroblast growth factor (bFGF)], and HIF-1α. The presence of MDSCs has been described in RCC patients and can account for their impaired immune response [35].

Therefore, understanding the mechanism and checkpoint regulators of MDSC-tumor interaction is critically important to overcome immunosuppression and to achieve better therapeutic effects in cancer patients.

### Dendritic cells

DCs are efficient antigen-presenting cells that can present tumor-specific antigens and subsequently activate a specific antitumor T cell response in vivo. Immature DCs process antigens and then mature to activated DCs, subsequently eliciting a productive immune response. DCs are not often found in tumor infiltrates [36], although sometimes they can be found in an immature state, unable to induce an effective immune response [37].

DCs have been shown to induce immune tolerance in several ways, including T cell deletion [38, 39], induction of T cell unresponsiveness [40] and Treg activation [41–43]. Immature DCs have been shown to silence immunity and to induce immune tolerance by inhibiting T cells or activating Treg cells [41] through tumor-derived factors, such as VEGF, IL-6, and macrophage colony-stimulating factor [44, 45].

Once activated, antigen-loaded DCs activate antigen-specific immunity [46], through T cell proliferation and differentiation into helper and effector cells. DCs also play an important role in humoral immunity, as they directly interact with B cells [47] and present unprocessed antigens [48].

Numerous studies in humans have concluded that DCs can infiltrate and fight tumors, through at least two pathways: directly via DC-dependent tumor cytokines and indirectly, via the induction of potent cytotoxic T-lymphocyte (CTL) responses.

In the context of RCC, it is important to highlight that carcinogenic cells have been described as inhibiting DC maturation, DC-induced T cell activation, and antitumor CTL response, by releasing IL-6 and VEGF [49]. Besides, increased tumor infiltration of DCs has been shown as a predictor for treatment response and an outcome in mRCC patients treated with immunotherapy [50].

### T regulatory cells

There is growing evidence that CD4^+^ CD5^+^ Treg cells may play an important role in suppressing the development of antitumor immunity in cancer patients [51] and that the number of Treg cells is increased in tumor sites and/or the peripheral blood of patients with advanced tumors [52–54].

### Natural killer cells

Natural killer (NK) cells are part of the hematopoietic system and are derived from CD34^+^ hematopoietic progenitor cells [55–57]. NK cells recognize and lyse tumor cells with no need for maturation or major histocompatibility complex recognition; besides, they are thought to be involved in immune surveillance against cancer [58, 59]. There is also some evidence of the critical role of NK cells in angiogenesis inhibition by IL-12-induced IFNγ secretion. In RCC terms, the presence of lymphocytic infiltrate with high levels of NK cells has been described as a good prognostic factor [60].

### Monocytes/macrophages

Monocytes/macrophages represent a quantitative and functionally important subpopulation within the tumor microenvironment. TAMs are derived from circulating peripheral blood monocytes attracted to the tumor site by chemotactic factors, such as colony stimulating factor 1, which also induce macrophage differentiation [61] and are involved in malignant progression. TAMs mediate their immunosuppressive activity by releasing inhibitory cytokines such as TGF-β and enhancing the production of IL-10 [62].

In addition, TAMs produce large amounts of VEGF and might be responsible for the tumor angiogenic switch [63]. In RCC, the number of TAMs significantly correlates with tumor microvessel density and VEGF levels [64].

## Immune dysfunction: type 1/type 2 bias

In RCC patients, there is a shift from a type-1-mediated CD4^+^ T cell response producing IFNγ, which is critical for the development of effective antitumor immunity, to a type-2 cytokine IL-4, IL-5, and IL-10 response that typically mediates humoral immunity [65].

Type-2 bias exists in T cells from mRCC patients, as tumor-specific CD4^+^ T cells displaying a T-helper type-2 bias have been isolated from mRCC patients [6, 7]. Additional studies have shown that a type-2 bias is present in T cells from mRCC patients after polyclonal stimulation of peripheral blood mononuclear cells (PBMC) with anti-CD3/anti-CD28 antibodies [8, 66, 67]. When compared with healthy donors, mRCC patients show a significant reduction in the percentage of CD4^+^ T cells expressing intracellular IFNγ, whereas the percentage of IL-4^+^ T cells was similar in both. Moreover, it has been demonstrated that RCC patients whose tumor environment is biased toward a type-1 immune response have a more favorable prognosis [68].

## Modulation of immune cells by tyrosine kinase inhibitors

Two kinds of antiangiogenic molecules are currently available: TKIs, which target receptors of proangiogenic factors and block their signaling functions; and monoclonal antibodies, which directly target circulating proangiogenic factors or their receptors.

Immune therapy usually requires time to generate, activate, and stimulate an antitumor immune response. Previous reports have described how antiangiogenic treatment could normalize tumor vessels as early as 2 days post-treatment [69, 70]. Therefore, the combined treatment schedule should be designed to synchronize vascular normalization and T cell activation. A previous study has suggested that antiangiogenic therapy preceding vaccine therapy has a better antitumor effect than vaccine therapy followed by antiangiogenic treatment [71]. Recently, immunotherapy with nivolumab showed longer overall survival than everolimus among patients with advanced RCC previously treated with antiangiogenic therapies [72]. A subgroup analysis of this last study found that patients previously treated with pazopanib showed a statistically significant increase in overall survival with nivolumab, while patients previously treated with sunitinib showed insignificant differences in overall survival between nivolumab and everolimus. This suggests that different TKIs might enhance subsequent immunotherapy by different mechanisms [73]. Thus, studies comparing the efficacy of different combination schedules might yield even better treatment regimens in the future.

The TKIs, sunitinib, pazopanib, sorafenib, and axitinib, have an impact not only on both tumor growth and angiogenesis, but also on the activity and function of immune effector cells. TKI treatment results in a dramatic reduction of T cell proliferation, along with distinct repercussions on cell cycle progression. Administration of TKIs reduces absolute neutrophil [74], monocyte [75], and lymphocyte counts [76, 77]. Of these, monocyte count undergoes the largest proportional decrease [78]. Patients with clinical benefit have a significantly smaller decrease in monocyte levels during the first cycle of treatment than patients with progressive disease [78]. A decrease in the absolute neutrophil count was associated with longer time to progression [79], while a decrease in total number of lymphocytes after sunitinib treatment has shown no benefit in terms of overall survival, when compared to patients with stable or increased total lymphocytes count [76]. Platelets also seem to play a role in the inmune response. Cysteine-rich protein 61(CYR61) connective tissue growth factor nephroblastoma overexpressed 1 (CCN1) is produced by endothelium cells and platelets and coats the internal side of the blood vessel. The increase of CCN1 amount is essential both for the recruitment of resident monocytes and for their patrolling activity. Without platelets in the blood, the CCN1 level will not rise and, therefore, the early arrival of Ly6C^low^ monocytes will be impaired and the recruitment of neutrophils abolished. Treatments that decrease the number of platelets may compromise the start of immune response [80]. This reduction in immune cells counts might explain, at least in part, some of the hematologic side effects of TKIs.

Targeting immunosuppressive cells might improve antitumor T cell response, thereby providing a rationale for the combination of TKIs with immunotherapy in the treatment of RCC.

Some studies suggest that Treg cells may be involved in modulating type-1 and type-2 cytokine response and that a reduction in their level is associated with an increase in T cell function as measured via IFN-γ levels [77]. Likewise, this increase in T cell function is also correlated with a reduction in MDSC populations, suggesting that both MDSCs and Treg cells are contributing to immune dysfunction in mRCC patients.

TKIs induce a reduction in Treg cell levels in mRCC patients. Their effect on Treg cell levels in peripheral blood has been examined in patients before sunitinib treatment and on day 28 of both first and second cycles. Compared to pretreatment values, the percentage of Treg cells was reduced after 1 cycle, although the reduction did not reach statistical significance. However, a negative correlation between the decreases of Treg cells after 1 or 2 cycles of treatment, and the increase of IFNγ-producing CD4^+^ T cells, was shown at the end of cycle 2. A negative correlation was also seen with the IFNγ response of total T cells (CD3^+^) population [77]. Thus, these findings may support the fact that the greater the reduction of Treg cells following 1 or 2 cycles of treatment, the stronger the type-1 response after 2 cycles of treatment [77].

The results reviewed here suggest that TKIs may have an effect on Treg cell population resulting in improved type-1 cytokine response. One plausible explanation for these findings is that the restoration of a type-1 IFNγ response is related to a decrease in the immunosuppressive tumor burden.

The hypothesis that the clinical response induced by TKIs is influenced by the degree of type-2 bias at baseline could be explained either by an immune mechanism of this therapeutic family or by the importance of parallel mechanisms of antitumor effect (TKI-induced antiangiogenic and innate immune response).

TKIs can reverse the immune suppression caused by Treg or MDSCs [77, 81, 82] and improve type 1 T cell cytokine response [77, 82–84], and these immunologic effects are currently under discussion. Moreover, the expression of negative co-stimulatory molecules, such as CTLA4 and PD-1 in CD4^+^ and CD8^+^ T cells, has also been shown to significantly decrease in sunitinib-treated mice [82], whereas a decrease of the T cell-mediated immune response has been observed upon sunitinib treatment in another murine model [85].

Some mechanisms that might explain the reduction of TKI-induced Treg cells, such as MDSC inhibition [81] or blockage of the conversion of conventional CD4^+^ Foxp3^−^ T cells into CD4^+^ Foxp3^+^ Treg cells [86], have been described.

TKIs seem to modulate MDSCs in different ways, such as: (1) inhibition of signal transducer and activator of transcription 3 (STAT3) [87], (2) induction of monocytic subset (Gr1^lo^) of MDSC proliferation, and (3) apoptosis of the granulocytic subset (Gr1^hi^) of MDSCs [34]. In mRCC patients, the proportion of all MDSC subsets (immature lin^−^, monocytic CD33^+^ CD14^+^ DR^−^ and granulocytic CD33^+^ CD15^+^ DR^−^) has been shown to decrease in peripheral blood after the first cycle of sunitinib treatment [81]. Similarly, only patients who do not respond to pazopanib treatment show increased MDSCs [84]. More importantly, MDSCs, either directly or through the induction of Treg cells, are involved in the development of TKI resistance [88].

TKIs could also regulate the expression of NK cell ligands, activating receptors in tumor cells. Thus, sunitinib and sorafenib induce NKG2D ligand expression, which confers enhanced sensitivity to NK cell lysis [89, 90]. Sorafenib also inhibits spontaneous and IL-2-induced NK effector functions [91], while axitinib has been shown to strongly suppress T cells proliferation, which might possibly affect the expansion of tumor-specific T cells [92]. In addition, axitinib enhances NK cell recognition and activity against RCC cells, by regulating NK cell-activating ligand expression [93].

Sorafenib inhibits DC antigen presentation and can stimulate primary T cell responses, by reducing the secretion of cytokines or the expression of MHC and CD1a molecules [94]. More importantly, its action in reversing the inhibitory effects of VEGF on monocyte-derived DC maturation and DC-mediated T cell stimulation has also been demonstrated [95]. The immunomodulatory effects of sorafenib on monocytes and macrophages have also been reported to induce autophagy and suppression of human macrophages [96].

Low doses of antiangiogenic treatment (sorafenib and sunitinib) have been shown to normalize tumor vasculature and polarize TAMs, thus reducing immune-regulatory signals and thereby creating an immune-supportive microenvironment for the recruitment and activatation of CD8+ T cells [89]. By this mechanism, low-dose antiangiogenic treatment enhances the anticancer efficacy of a vaccine therapy. In contrast, high-dose antiangiogenic treatment excessively reduces tumor vessels, thus decreasing the delivery of chemotherapeutics [97, 98]. High-dose antiangiogenic treatment may also exacerbate, rather than reverse, the immunosuppressive tumor microenvironment, thus compromising the efficacy of active cancer immunotherapy. Therefore, these studies suggest that appropriate low-dose antiangiogenic treatment could be an effective strategy to reengineer the tumor microenvironment for active immunotherapies in a clinical setting.

## Conclusions and future perspective

The existence of several tumor-mediated immunosuppressive networks operating in RCC that impede the success of immune-based therapies is now widely accepted. One of them involves tumor-induced accumulation of MDSCs [99].

Emerging data indicate that abnormal tumor vasculature, resulting from the prevalence of proangiogenic factors over antiangiogenic signals, fosters an immunosuppressive tumor microenvironment, which in turns enables host immunosurveillance evasion by the tumor. Proangiogenic factors not only suppress the function of various immune cells but also diminish leukocyte–endothelial interactions and hinder the infiltration of immune effector cells into the tumor parenchyma.

The induction of high levels of tumor-specific cytotoxic T lymphocytes is a prerequisite for successful cancer immunotherapy. Unfortunately, the presence of a high number of tumor antigen-specific cytotoxic T cells in peripheral immune organs is associated with little clinical benefit, so other factors, such as tumor microenvironment, considered a key player, must be involved in this poor clinical outcome.

Immunotherapy of mRCC has evolved from a rather unspecific (e.g. cytokine era) to a more specific (e.g. checkpoint inhibitors) approach. Immunological checkpoints can either inhibit or activate T cells, leading to speculation as to whether immunomodulation should target T cell-inhibiting or T cell-activating co-stimulatory molecules. The fact that TKIs can improve type-1 T cell cytokine response while reducing both number and function of Treg cells, provides a basis for the rational combination of TKIs and immunotherapy in mRCC.

This mechanism is currently under investigation, although it is likely to be in part due to MDSC reduction which inhibits T cell function directly.

Administration of TKIs before vaccination induces higher antitumor efficacy than post-vaccination or simultaneous administration. The modulation of tumor-induced immunosuppression, which results in a better induction of antigen-specific CD8^+^ T cells after vaccination, could explain this synergy. TKIs could normalize tumor vascularization transiently, thus helping CD8^+^ T cell influx into the tumor after vaccination. Vascular normalization could also be accompanied by decreased hypoxia. Since hypoxia seems to be involved in the development of immunosuppressive mechanisms, its suppression could represent a mechanism for modulating tumor-induced immunosuppression.

CheckMate-025 sub-analyses revealed potential outcome differences with nivolumab following pazopanib and sunitinib, showing only statistically significant increase in the overall survival only in the case of pazopanib. Taking this into account, analyzing tumor samples from the PISCES trial would be of great interest to study whether pazopanib is a better primer compared to sunitinib, before a second line immunomodulator.

To summarize, rationally scheduled antiangiogenic treatment can transiently normalize tumor vessels, improve vessel perfusion, decrease hypoxia and enhance the efficacy of cytotoxic therapies.
